# A first-in-man phase 1 study of the DNA-dependent protein kinase inhibitor peposertib (formerly M3814) in patients with advanced solid tumours

**DOI:** 10.1038/s41416-020-01151-6

**Published:** 2020-11-24

**Authors:** Mark T. J. van Bussel, Ahmad Awada, Maja J. A. de Jonge, Morten Mau-Sørensen, Dorte Nielsen, Patrick Schöffski, Henk M. W. Verheul, Barbara Sarholz, Karin Berghoff, Samer El Bawab, Mirjam Kuipers, Lars Damstrup, Ivan Diaz-Padilla, Jan H. M. Schellens

**Affiliations:** 1 Department of Clinical Pharmacology, The Netherlands Cancer Institute, Amsterdam, The Netherlands; 2 Oncology Medicine Department, Jules Bordet Institute, Université Libre de Bruxelles, Brussels, Belgium; 3 Medical Oncology, Erasmus MC Cancer Institute, Rotterdam, The Netherlands; 4 Department of Oncology, Rigshospitalet, Copenhagen Denmark; 5 Department of Oncology, Herlev and Gentofte Hospital, Herlev, Denmark; 6 Department of General Medical Oncology, Leuven Cancer Institute, University Hospitals Leuven, Leuven, Belgium; 7 Department of Medical Oncology, Radboud University Medical Center, Nijmegen, The Netherlands; 8 Merck KGaA, Darmstadt, Germany; 9 Ares Trading S.A., Eysins, Switzerland; an Affiliate of Merck KGaA, Darmstadt, Germany; 10 Present Address: Debiopharm International S.A., Lausanne, Switzerland; 11 Present Address: Synthon Biopharmaceuticals, Nijmegen, The Netherlands

**Keywords:** Phase I trials, Drug development

## Abstract

**Background:**

This open-label, phase 1 trial (NCT02316197) aimed to determine the maximum-tolerated dose (MTD) and/or recommended phase 2 dose (RP2D) of peposertib (formerly M3814), a DNA-dependent protein kinase (DNA-PK) inhibitor in patients with advanced solid tumours. Secondary/exploratory objectives included safety/tolerability, pharmacokinetic/pharmacodynamic profiles and clinical activity.

**Methods:**

Adult patients with advanced solid tumours received peposertib 100–200 mg once daily or 150–400 mg twice daily (BID) in 21-day cycles.

**Results:**

Thirty-one patients were included (median age 66 years, 61% male). One dose-limiting toxicity, consisting of mainly gastrointestinal, non-serious adverse events (AEs) and long recovery duration, was reported at 300 mg BID. The most common peposertib-related AEs were nausea, vomiting, fatigue and pyrexia. The most common peposertib-related Grade 3 AEs were maculopapular rash and nausea. Peposertib was quickly absorbed systemically (median *T*_max_ 1.1–2.5 h). The p-DNA-PK/t-DNA-PK ratio decreased consistently in peripheral blood mononuclear cells 3–6 h after doses ≥100 mg. The best overall response was stable disease (12 patients), lasting for ≥12 weeks in seven patients.

**Conclusions:**

Peposertib was well-tolerated and demonstrated modest efficacy in unselected tumours. The MTD was not reached; the RP2D was declared as 400 mg BID. Further studies, mainly with peposertib/chemo-radiation, are ongoing.

**Clinical trial registration:**

NCT02316197

## Background

DNA double-strand breaks (DSBs) are the most cytotoxic type of DNA lesions, which if left unrepaired, can cause cell-cycle arrest via checkpoint activation and subsequent cell death.^1^ Complex signalling pathways, collectively termed the DNA damage response (DDR), exist within cells to detect and repair DNA damage, including DNA DSBs.^1^ Defects in DDR pathways can lead to the accumulation of non-lethal DNA damage and genomic instability, which is a hallmark of many cancers and drives tumourigenesis.^2^

Many anti-cancer treatments, such as radiotherapy and some chemotherapies, work by inducing DNA DSBs in tumour cells. The efficacy of such treatments can be compromised by the efficient repair of DNA damage through activation of the DDR, including DNA-dependent protein kinase (DNA-PK).^3^ DNA-PK is a serine/threonine protein kinase that plays a critical role in the DDR and regulates DNA DSB repair via the non-homologous end joining (NHEJ) pathway.^4,5^

Peposertib (formerly M3814) is an orally administered, small-molecule, selective DNA-PK inhibitor that blocks DNA-PK kinase activity at sub-nanomolar concentrations, inhibiting its ability to function in the DNA repair process leading to the persistence of DNA DSBs and subsequent cell death.^6–8^ Whilst peposertib has previously demonstrated selectivity for several members of the phosphoinositide 3 kinase (PI3K) and PI3K-related family of proteins, of which DNA-PK, ataxia-telangiectasia-mutated (ATM) and ataxia-telangiectasia and Rad3-related (ATR) kinases are members, its potency against DNA-PK was substantially greater than against other family members.^8^ Additionally, peposertib demonstrated synergy with radiotherapy and DSB-inducing chemotherapies (e.g. etoposide, doxorubicin) in preclinical studies by preventing the repair of radiation- or chemotherapy-induced DNA DSBs via the NHEJ pathway.^6–10^

The purpose of this first-in-man, open-label, phase 1 trial (NCT02316197) was to determine the maximum-tolerated dose (MTD) and/or the recommended phase 2 dose (RP2D) of peposertib. To our knowledge, this is the first full report of an oral DNA-PK inhibitor in humans. The results of this study have been partially presented at the American Society for Clinical Oncology Annual Meeting in 2017^11^ and at the European Society for Medical Oncology Annual Meeting in 2018.^12^

## Methods

### Trial design

This phase 1, first-in-man, open-label, dose-escalation study (NCT02316197) was designed to explore the safety, tolerability, pharmacokinetic (PK) and pharmacodynamic (PD) profiles, and clinical activity of peposertib administered daily as a single agent to patients with advanced solid tumours likely to have alterations in DNA repair mechanisms. Following screening and baseline evaluations, patients received peposertib until disease progression, unacceptable toxicity, withdrawal of consent or other reasons necessitating withdrawal (Supplementary Fig. S1).

Dose escalation was by use of a standard ‘3 + 3’ design, based on the presence/absence of dose-limiting toxicities (DLTs). Patients in the first dose cohort received peposertib continuously in 21-day cycles at a starting dose of 100 mg once daily (QD). The starting dose was considered to have acceptable safety and to be in the range of the biologically active dose based on in vivo pharmacology data.^7^ Patients in the second cohort received peposertib at a dose of 200 mg QD. Subsequent cohorts received ascending doses of peposertib as follows: 150 mg twice daily (BID); 200 mg BID; 300 mg BID; and 400 mg BID.

The study was conducted in accordance with the ethical principles of the International Council for Harmonisation guideline for Good Clinical Practice and the Declaration of Helsinki, as well as with applicable local regulations.

### Patients

Patients aged ≥18 years with advanced solid tumours, for whom no other standard surgical, radiation or systemic anti-cancer therapies were available, and with tumour accessible for biopsies, and measurable or evaluable disease by Response Evaluation Criteria in Solid Tumours (RECIST) v1.1^13^ were eligible for inclusion in the study. Although not mandatory, the intention was to select patients based on the likelihood of alterations in DNA repair mechanisms (triple-negative breast, serous epithelial ovary, bladder, microsatellite instability-high colon, lung, castration-resistant prostate, stomach or uterine cancer). Patients were excluded from the study if they had an Eastern Cooperative Oncology Group performance status (ECOG PS) > 1; had received any anti-cancer therapy (except for luteinising hormone-releasing hormone analogues) or any other investigational agent within 28 days of the first dose of peposertib; had received extensive radiotherapy on >30% of bone marrow reserves or prior bone marrow/stem cell transplantation within 5 years of study start; or were receiving medications/herbal supplements known to be potent inhibitors or inducers of CYP3A or CYP2C19. All patients provided written informed consent.

### Study objectives and endpoints

The primary objective was to determine the MTD and/or the RP2D of peposertib assessed as the proportion of patients who experienced at least one DLT during the first 21-day treatment cycle. The MTD was defined as the dose level below the dose at which two out of six patients experienced a DLT. Once the MTD was established, the RP2D was to be defined by the Safety Monitoring Committee (SMC), either at the MTD level or below, depending on the available safety, efficacy, PK and PD data.

A DLT was defined as any of the following toxicities considered to be possibly related to peposertib by the sponsor and/or investigator during Cycle 1: a treatment-emergent adverse event (TEAE) of potential clinical significance such that further dose escalation would expose patients to unacceptable risk; evidence of possible treatment-related hepatocellular injury for ≥3 days; any Grade 4 liver enzyme elevation; any Grade ≥3 toxicity (defined according to National Cancer Institute Common Terminology Criteria for Adverse Events v4.03,^14^ and excluding diarrhoea, nausea and vomiting of <3 days duration following adequate therapy, Grade 3 skin toxicity resolving to Grade ≤2 with supporting measures within 7 days, fatigue or headache of <7 days duration following initiation of adequate supportive care, any other single laboratory value out of the normal range that was not correlated to clinically significant symptoms, Grade 3 thrombocytopenia without bleeding, neutropenia lasting for ≤5 days and not associated with fever); any toxicity related to study drug resulting in ≥20% of the planned dose to be missed in Cycle 1. In addition, a DLT could be identified by the SMC as any TEAE that impaired daily function or abnormality occurring in patients treated with peposertib at any time during the dose escalation part of the study. TEAEs were defined as adverse events (AEs) observed from the first dose of peposertib to 30 days after the last dose, whilst a serious TEAE was defined as any TEAE that was life threatening, required hospitalisation or prolongation of existing hospitalisation, resulted in death, or was otherwise considered as medically important.

Secondary objectives included evaluation of the safety and tolerability of peposertib, assessment of PK and exploration of anti-tumour activity. Safety was assessed through the recording, reporting and analysis of baseline medical conditions, AEs, physical examination findings (including vital signs and monitoring for bleeding), laboratory tests, ECOG PS and 12-lead electrocardiograms. Based on preclinical data showing reversible inhibition of platelet aggregation at micromolar concentrations, the potential impact of peposertib on human cyclooxygenase 1 was investigated by evaluating the inhibition of platelet aggregation in six aspirin-naïve patients treated at the RP2D. PK was assessed using blood samples collected pre-treatment and on-treatment. The following PK parameters were evaluated: maximum observed concentration (*C*_max_); dose-normalised *C*_max_ (*C*_max_/dose); time to *C*_max_ (*t*_max_); area under the plasma concentration–time curve (AUC) from 0 to 12 h (AUC_0–12_); dose-normalised AUC_0–12_ (AUC_0–12_/dose); AUC from 0 to infinity (AUC_0–∞_; day 1 only); apparent elimination half-life; apparent total body clearance of drug; apparent total body clearance at steady state of drug (cycle 2 only); apparent volume of distribution during terminal phase (*V*_z/f_); accumulation ratio for AUC (*R*_acc(AUC)_); and accumulation ratio for *C*_max_ (*R*_acc(Cmax)_). Tumour response was evaluated according to RECIST (v1.1). Target and non-target lesions were measured by computed tomography or magnetic resonance imaging every 6 weeks.

Exploratory objectives included assessing the PD in peripheral blood mononuclear cells (PBMCs), isolated from serial blood samples collected pre-treatment and on-treatment. PBMCs were exposed to the DNA-damaging agent bleomycin to induce DNA-PK activity ex vivo. The level of the autophosphorylated form of DNA-PK on Ser^2056^ (p-DNA-PK) was assessed as a PD biomarker for peposertib. Phospho-DNA-PK levels normalised to total DNA-PK (t-DNA-PK) were measured using the Erenna® Immunoassay System (Singulux, Alameda, California, USA) in PBMC lysates, and calculated as the p-DNA-PK concentration (ng/mL)/t-DNA-PK concentration (ng/mL). DNA-PK inhibition by peposertib on-treatment was expressed as the percentage change of the PD biomarker versus the pre-treatment value.

### Analyses

The cut-off for the final analysis was when the last patient completed the dose-escalation phase (29 June 2017). The All Patients Analysis Set comprised all patients who provided informed consent (screening failures plus enrolled patients) and was used to describe patient disposition and deaths. The Safety Analysis Set included patients who received at least one dose of peposertib and was used for all baseline and safety (except for DLTs) summaries. The Efficacy Analysis Set, including patients who received at least one treatment dose, was used for all efficacy summaries. The Dose-Escalation Analysis Set included patients treated in dose-escalation cohorts who received at least 80% of peposertib planned doses in the first treatment cycle or who experienced a DLT during the first treatment cycle regardless of the amount of drug received (used for DLT summary). The PK Analysis Set (cycles 1 and 2) included patients who received at least the first dose of peposertib and provided PK samples as per protocol for at least 24 h following the first dose on day 1 of cycle 1 for the QD regimen, or for at least 12 h following first dose on day 1 of cycle 1 for the BID regimen. The Biomarker/Pharmacogenomics Analysis Set included patients who received at least the first dose of study drug and provided at least one pre-dose sample and one post-dose sample.

TEAEs were summarised according to Medical Dictionary for Regulatory Activities (v20.0) preferred term and system organ class. All statistical analyses (except for PK) were performed using SAS 9.1.3 or higher. Tumour assessments, based on investigator evaluations of target, non-target and new lesions according to RECIST (v1.1), were used to derive the best overall response. Trial data were summarised by dose level.

## Results

### Patient baseline demographics and disease characteristics

**Fig. 1:**
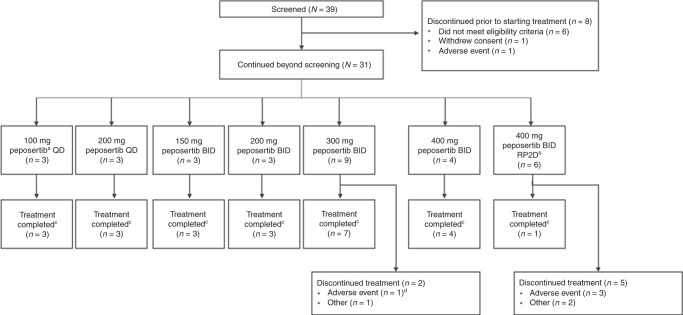
Patient disposition. ^a^Formerly M3814; ^b^The six patients in the RP2D cohort were aspirin-naïve; ^c^Patients were treated until disease progression or death; ^d^DLT, due to a combination of low grade, mainly gastrointestinal, non-serious AEs and the long duration of recovery following treatment discontinuation. BID twice daily, DLT dose-limiting toxicity, QD once daily, RP2D recommended phase 2 dose.

Patient disposition is shown in Fig. 1. Of the 39 patients who were screened, eight patients did not receive peposertib: six did not meet the eligibility criteria, one withdrew consent and one did not continue beyond screening due to a serious AE (intestinal obstruction). Thirty-one patients were then included in the study (100 mg QD, *n* = 3; 200 mg QD, *n* = 3; 150 mg BID, *n* = 3; 200 mg BID, *n* = 3; 300 mg BID, *n* = 9; 400 mg BID, *n* = 4; 400 mg BID [declared as the RP2D], aspirin-naïve, *n* = 6). The numbers of patients included in each of the analysis sets were as follows: Safety/Efficacy Analysis Set, *n* = 31; Dose-Escalation Analysis Set, *n* = 21; PK Analysis Set, *n* = 31; Biomarker Analysis Set, *n* = 31. The first patient’s first visit was on 14 January 2015 and the last patient’s last visit was on 29 June 2017.

Patient baseline characteristics are presented in Table 1. The median (range) age was 66 (25–78) years; 19/31 (61%) patients were male; 27/31 (87%) were Caucasian; ECOG PS at baseline was 0 in ten (32%) patients and 1 in 68% of patients.

**Table 1:** Baseline patient demographics and disease characteristics.

Parameter	Categories/characteristics	Total^a^
Sex, *n* (%)	Male	19 (61)
	Female	12 (39)
Race, *n* (%)	Caucasian	27 (87)
	Other	4 (13)
Age (years)	Mean (SD) Median (range)	62 (12) 66 (25–78)
ECOG PS, *n* (%)	0 1	10 (32) 21 (68)
Primary tumour location (tumour type added where applicable), *n* (%)	Colon/rectum (colorectal) Liver (hepatocarcinoma) Skin (malignant melanoma) Lung (NSCLC) Bone (osteosarcoma) Parotid gland Head/neck (SCCHN) Adrenal Bladder Breast Bile duct (cholangiocarcinoma) Thigh (liposarcoma) Ovary Pancreas Kidney (renal cell cancer) Uterus Other^b^	9 (29) 2 (7) 2 (7) 2 (7) 2 (7) 2 (7) 2 (7) 1 (3) 1 (3) 1 (3) 1 (3) 1 (3) 1 (3) 1 (3) 1 (3) 1 (3) 1 (3)

*ECOG PS* Eastern Cooperative Oncology Group performance status, *NSCLC* non-small cell lung cancer, *SCCHN* squamous cell carcinoma of the head and neck, *SD* standard deviation.

^a^Percentages may not total 100% due to rounding.

^b^Skin or other parts of the face.

The majority of patients were treated until disease progression or death, except for two patients in the 300 mg BID cohort (one discontinued due to an AE [DLT, due to a combination of low-grade, mainly gastrointestinal, non-serious AEs and the long duration of recovery following treatment discontinuation] and the other due to patient request after experiencing fatigue (Grade 3) amongst other Grade ≤2 AEs), and five patients in the 400 mg BID (RP2D) cohort who discontinued due to AEs (*n* = 3; colitis in one patient [unrelated to peposertib]; maculopapular rash in one patient [related to peposertib]; nausea and papular rash in one patient [both related to peposertib]; all were considered serious and all were Grade 3) and clinical progressive disease (*n* = 2).

### Dose-limiting toxicities and maximum-tolerated dose

Safety evaluation was based on peposertib exposure of up to a maximum of 484 days (69 weeks). One DLT was reported for one patient in the 300 mg BID cohort: no individual AE qualified as a DLT; however, a combination of non-serious AEs (stomatitis, decreased appetite, dysgeusia, erythema, urticaria, fatigue and nausea) and long recovery duration following treatment discontinuation was considered as a DLT. The MTD of peposertib was not reached at 400 mg BID and dose escalation was stopped at 400 mg BID. As there were no DLTs reported at this dose level, 400 mg BID was declared as an acceptable RP2D. This RP2D was further supported by the exposure levels observed at 400 mg BID to be in the expected range of target engagement.^8^ Six aspirin-naïve patients (see above) were enrolled at the RP2D to confirm the safety and tolerability and to the effects of peposertib in platelet aggregation. The lack of objective responses or clearly identifiable efficacy signals in the patients treated precluded the initiation of the dose-expansion cohort of the study.

### Safety

The median (range) treatment duration with peposertib was 6.0 (0.3–69.0) weeks. All 31 patients had at least one TEAE, and most (71%) had at least one TEAE related to peposertib. Nausea (*n* = 17), fatigue (*n* = 14), pyrexia (*n* = 11) and constipation (*n* = 10) were the most common TEAEs. Nausea (*n* = 8), vomiting (*n* = 6), fatigue (*n* = 6) and pyrexia (*n* = 5) were the most frequently reported TEAEs related to peposertib (Table 2). Twenty-one patients (68%) had at least one TEAE of Grade ≥3. Peposertib-related Grade 3 TEAEs occurred in seven (23%) patients. The most common Grade 3 peposertib-related TEAEs were maculo-papular rash (*n* = 4) and nausea (*n* = 2). There were no peposertib-related Grade 4 TEAEs.

**Table 2:** Most common treatment-related TEAEs by highest grade.

Peposertib^a^ dose (mg)/frequency of administration									
Preferred term *n* (%)	Grade^b^	100/QD *n* = 3	200/QD *n* = 3	150/BID *n* = 3	200/BID *n* = 3	300/BID *n* = 9	400/BID *n* = 4	400/BID (RP2D) *n* = 6	Total *N* = 31
Patients with ≥ 1 event		1 (33)	2 (67)	2 (67)	0 (0)	8 (89)	3 (75)	6 (100)	22 (71)
Nausea	Any	0 (0)	0 (0)	0 (0)	0 (0)	2 (22)	2 (50)	4 (67)	8 (26)
	Grade 3	0 (0)	0 (0)	0 (0)	0 (0)	0 (0)	1 (25)	1 (17)	2 (7)
Vomiting	Any	0 (0)	0 (0)	0 (0)	0 (0)	2 (22)	2 (50)	2 (33)	6 (19)
	Grade 3	0 (0)	0 (0)	0 (0)	0 (0)	0 (0)	0 (0)	0 (0)	0 (0)
Fatigue	Any	0 (0)	0 (0)	1 (33)	0 (0)	5 (56)	0 (0)	0 (0)	6 (19)
	Grade 3	0 (0)	0 (0)	0 (0)	0 (0)	1 (11)	0 (0)	0 (0)	1 (3)
Pyrexia	Any	0 (0)	0 (0)	0 (0)	0 (0)	1 (11)	1 (25)	3 (50)	5 (16)
	Grade 3	0 (0)	0 (0)	0 (0)	0 (0)	0 (0)	1 (25)	0 (0)	1 (3)
Stomatitis	Any	0 (0)	0 (0)	0 (0)	0 (0)	2 (22)	1 (25)	1 (17)	4 (13)
	Grade 3	0 (0)	0 (0)	0 (0)	0 (0)	0 (0)	1 (25)	0 (0)	1 (3)
Maculopapular rash	Any	0 (0)	0 (0)	0 (0)	0 (0)	1 (11)	1 (25)	2 (33)	4 (13)
	Grade 3	0 (0)	0 (0)	0 (0)	0 (0)	1 (11)	1 (25)	2 (33)	4 (13)
Constipation	Any	0 (0)	1 (33)	0 (0)	0 (0)	2 (22)	0 (0)	0 (0)	3 (10)
	Grade 3	0 (0)	0 (0)	0 (0)	0 (0)	0 (0)	0 (0)	0 (0)	0 (0)
Diarrhoea	Any	0 (0)	1 (33)	1 (33)	0 (0)	0 (0)	0 (0)	1 (17)	3 (10)
	Grade 3	0 (0)	0 (0)	0 (0)	0 (0)	0 (0)	0 (0)	0 (0)	0 (0)
Rash	Any	0 (0)	0 (0)	0 (0)	0 (0)	2 (22)	0 (0)	1 (17)	3 (10)
	Grade 3	0 (0)	0 (0)	0 (0)	0 (0)	1 (11)	0 (0)	1 (17)	2 (7)
Periorbital oedema	Any	0 (0)	0 (0)	0 (0)	0 (0)	1 (11)	0 (0)	1 (17)	2 (7)
	Grade 3	0 (0)	0 (0)	0 (0)	0 (0)	0 (0)	0 (0)	0 (0)	0 (0)
Dry mouth	Any	0 (0)	0 (0)	0 (0)	0 (0)	2 (22)	0 (0)	0 (0)	2 (7)
	Grade 3	0 (0)	0 (0)	0 (0)	0 (0)	0 (0)	0 (0)	0 (0)	0 (0)
Decreased appetite	Any	0 (0)	0 (0)	1 (33)	0 (0)	1 (11)	0 (0)	0 (0)	2 (7)
	Grade 3	0 (0)	0 (0)	0 (0)	0 (0)	0 (0)	0 (0)	0 (0)	0 (0)

Medical Dictionary for Regulatory Activities v20.0.

*BID* twice daily, *QD* once daily, *RP2D* recommended phase 2 dose, *TEAE* treatment-emergent adverse event.

^a^Formerly M3814.

^b^No treatment-related Grade 4 or 5 TEAEs were observed in any treatment group.

Seventeen patients (55%) had a serious TEAE, most of which were Grade ≤3. However, two patients treated with peposertib 400 mg BID reported Grade 4 TEAEs. Peposertib-related serious TEAEs occurred in four (13%) patients, all at the highest dose level of 400 mg BID. Peposertib-related serious TEAEs were maculopapular rash in two patients; pyrexia and maculopapular rash in one patient; and nausea and maculopapular rash in one patient. Three patients died due to disease progression. One of these patients in the 400 mg BID cohort had a TEAE leading to death (Grade 5 general physical health deterioration considered unrelated to peposertib). None of the deaths were considered treatment-related (Supplementary Table S1).

In the aspirin-naïve cohort (400 mg BID, RP2D) a very weak effect on platelet aggregation was observed, which was not considered a safety signal.

### Pharmacokinetics

**Fig. 2:**
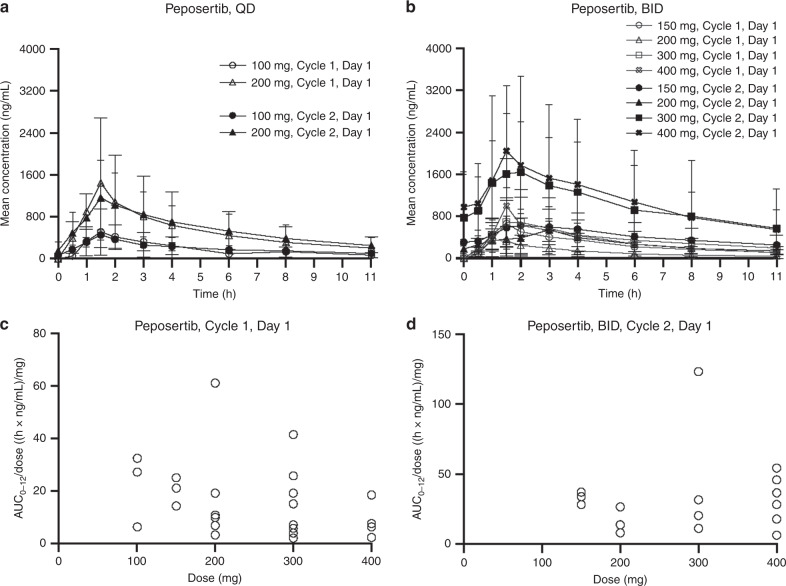
Peposertib (formerly M3814) pharmacokinetics. Plasma concentration–time profiles of peposertib (formerly M3814) following **a** QD (PK Analysis Set; *n* = 6) and **b** BID (PK Analysis Set; *n* = 25 [Cycle 1], *n* = 17 [Cycle 2]) dosing (linear scale). Dose-normalised AUC_0–12_ for peposertib by dose on **c** Cycle 1 Day 1 (QD and BID; PK Analysis Set; *n* = 25) and **d** Cycle 2 Day 1 (BID; PK Analysis Set; *n* = 17). AUC_0–12_ area under the plasma concentration–time curve from 0 to 12 h, BID twice daily, PK pharmacokinetics, QD once daily.

Following oral administration, peposertib was quickly absorbed into the systemic circulation with a median *t*_max_ of 1.1–2.5 h. The exposure parameters of peposertib showed high inter-individual variability across all doses as indicated by moderate to high geometric mean percent coefficient of variation (%CV) values (15.5–117% for *C*_max_; 12.5–124% for AUC_0–12_). Minimal accumulation of peposertib after QD and BID dosing was observed on cycle 2 day 1, following multiple dosing, in line with the observed mean elimination half-life of ~5.5 h (Fig. 2 and Supplementary Table S2). Plasma exposure of peposertib increased with increasing doses. However, high inter-individual variability in dose-normalised exposure prevented conclusive estimation of dose-proportionality (Fig. 2 and Supplementary Table S2).

### Pharmacodynamics

**Fig. 3:**
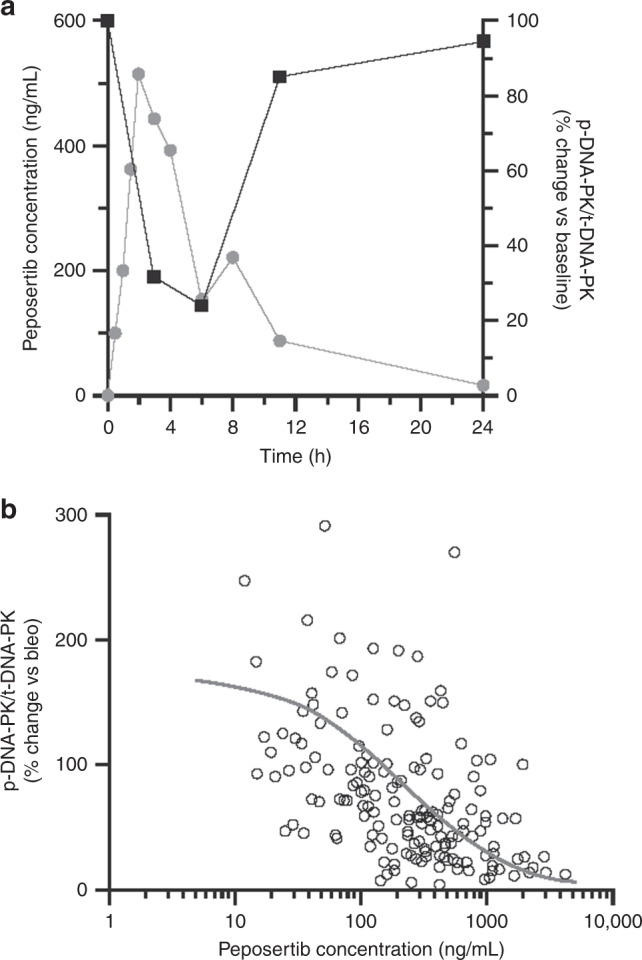
DNA-PK inhibition by peposertib (formerly M3814). In **a** typical time-course profiles of PK (grey circles) and DNA-PK inhibition (black squares) after peposertib treatment from one patient treated with peposertib 100 mg QD. DNA-PK inhibition on treatment is expressed as percentage change of p-DNA-PK/t-DNA-PK from the pre-treatment value (time *h* = 0). In **b** concentration-dependent inhibition of DNA-PK in response to peposertib treatment. Experimental concentration−response data are shown (open circles) and the line represents the *E*_max_ model fit to the data. Bleo bleomycin, DNA-PK DNA-dependent protein kinase, *E*_max_ maximum response, p-DNA-PK autophosphorylated form of DNA-PK on Ser^2056^, PK pharmacokinetics, QD once daily, t-DNA-PK total DNA-PK.

Overall, a consistent and marked dose-dependent decrease in the PD biomarker level (p-DNA-PK/total DNA-PK ratio) was observed in PBMCs, as a surrogate of tumour tissue, at early time points (3 and 6 h) after peposertib administration (Fig. 3). A typical PK profile was shown in one patient treated at 100 mg (Fig. 3a). In addition, peposertib concentration-dependent p-DNA-PK inhibition could be clearly established (Fig. 3b) with half-maximal inhibition (IC_50_) observed at peposertib concentrations of approximately 200 ng/mL (95% confidence intervals, 149–286). These results demonstrate that peposertib inhibits DNA-PK in a time- and concentration-dependent manner, providing evidence of target engagement at doses below the MTD.

### Efficacy

**Fig. 4:**
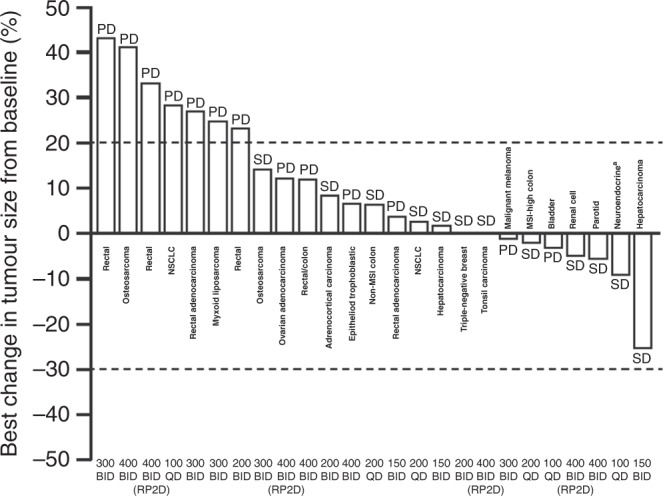
Waterfall plot showing best percentage change in tumour size from baseline and best overall response. Peposertib (formerly M3814) dose (mg)/frequency of administration is shown for each patient on the *x* axis. Dashed lines show the 20% threshold for PD and the 30% threshold for partial response. ^a^Colon. BID twice daily, MSI microsatellite instability, NSCLC non-small cell lung cancer, PD progressive disease, QD once daily, RP2D recommended phase 2 dose, SD stable disease.

No objective tumour responses (complete or partial) were reported. However, 12 patients (39%) had a best overall response of stable disease, of whom seven experienced prolonged stable disease for ≥12 weeks. Of note, one patient with a metastatic renal cell carcinoma who previously progressed on sunitinib, pazopanib, axitinib and everolimus, and another patient with a tonsillar squamous cell carcinoma who previously progressed on prior cisplatin-based chemotherapy at the maximum administered dose, 400 mg BID, remained on treatment for 36 weeks. Remarkably, a patient with a metastatic hepatocellular carcinoma and prior progression on sorafenib had stable disease for 69 weeks at the time of data cut-off. Five patients (16%) were not evaluable (tumour assessment at screening only) (Fig. 4).

## Discussion

Here, we report the first-in-human study evaluating a DNA-PK inhibitor in patients with advanced cancer. Single-agent peposertib, a potent and selective inhibitor of the DNA-PK catalytic activity,^6–10^ was well-tolerated when given orally QD or BID to patients with advanced solid tumours. The maximum administered dose was 400 mg BID, and this was also declared the RP2D in monotherapy, based on the achieved predicted exposure−efficacy relationship, and evidence of target engagement. It was however decided not to conduct the dose-expansion part of the study as it was considered that the early efficacy signals were modest. This represents a limitation of the study because no early signs of efficacy could be ascertained in a tumour-specific or in a biomarker-selected patient population. From a clinical safety perspective, this study did not reveal any unexpected safety signals, in line with the preclinical toxicology data available at the time of study initiation. The only DLT reported consisted of a combination of low-grade, non-serious, mostly gastrointestinal AEs in one patient treated with 300 mg peposertib BID.

Peposertib exhibits a high degree of selectivity when tested using a broad panel of 326 serine/threonine, tyrosine and lipid kinases. PI3K kinases, ATM, ATR, mammalian target of rapamycin and DNA-PK, are all members of the PI3K-related kinase family, which is characterised by high similarity in the kinase domain. Peposertib inhibited PI3K lipid kinases with significantly lower potency as demonstrated in preclinical studies.^8^ The inhibition of PI3K has been associated with some notable clinical AEs, such as hyperglycaemia, diarrhoea or rash.^15^ In this study, maculopapular rash was also reported in four patients. This was an intriguing clinical finding, as the preclinical experiments with peposertib showed only moderate inhibition of the phosphorylation of AKT, which is consistent with its weak inhibitory activity on the PI3K isoforms.^8^ Further data will be required to confirm any association between the occurrence of rash and the degree of PI3K inhibition elicited by peposertib in the clinical setting.

The present study also included a PD assessment of target engagement by peposertib in the clinical setting. Preclinical studies have demonstrated the feasibility of measuring DNA-PK inhibition by peposertib in human PBMCs, as an ex vivo surrogate of tumour tissue. The PD results of this study showed a concentration-dependent suppression of the phosphorylated fraction of DNA-PK indicating effective target inhibition by peposertib. This conclusion is limited to peripheral PBMCs since target engagement in paired-tumour biopsies were not collected.

To date, the use of DNA-PK inhibitors in clinical studies has been limited by the bioavailability of currently available molecules.^16^ Here, we show that the peposertib powder-in-capsule formulation was quickly absorbed into the systemic circulation, with slight accumulation observed with multiple QD and BID dosing. Peposertib plasma exposure increased with increasing dose, but dose proportionality could not be evaluated due to the large inter-patient variability in exposure. In addition, peposertib showed only a weak effect on platelet aggregation and this was not considered to be a safety signal. These findings do not support the exclusion of patients taking oral anti-coagulation medication from receiving peposertib treatment.

The lack of partial responses with peposertib monotherapy according to RECIST (v1.1) is not entirely unexpected. The current study was performed in unselected patients, in whom the genetic background of the tumours evaluated was unknown; therefore, mechanisms of synthetic lethality associated with DNA-PK inhibition could not be exploited. Due to the lack of objective efficacy results, with no patients achieving a best response of partial or complete response, the dose-expansion cohort of this study which would have included genetic and molecular screening was not initiated. However, preclinical reports suggest that certain molecular aberrations in tumours, such as ATM loss, may lead to increased sensitivity to DNA-PK inhibition.^17,18^ In addition, peposertib has been shown to inhibit DNA DSB repair and enhance existing sensitivity to radiation in cancer cells through inhibition of DNA-PK kinase activity.^6–9^ Preclinical results indicate that combinations of peposertib with DNA DSB-inducing agents such as radiotherapy and certain chemotherapeutic agents (e.g. topoisomerase 2 inhibitors) are strongly synergistic and more likely to yield tumour responses.^6,8,10^ To this end, there are currently two ongoing clinical trials evaluating peposertib in combination with (chemo-) radiotherapy. A phase 1a/1b study is testing the administration of peposertib given with palliative radiation in metastatic solid tumours, as well as the combination of peposertib with high-dose cisplatin and high-dose radiation in locally advanced squamous cell carcinoma of the head and neck (NCT02516813). The second study is a phase 1b/2 study evaluating the combination of peposertib with capecitabine and radiation for the neoadjuvant treatment of locally advanced rectal cancer (NCT03770689).

In conclusion, this first-in-human study of peposertib provides clinical evidence that peposertib inhibits DNA-PK activity and is well-tolerated when given orally as a single agent in doses up to 400 mg BID. Currently ongoing studies in combination with chemo-radiation should shed light as to whether the addition of peposertib could enhance the efficacy of standard therapeutic regimens in selected tumour types, and therefore having the potential to become a new therapeutic option for patients with cancer.

## Supplementary information

Supplementary material

## Data Availability

Any requests for data by qualified scientific and medical researchers for legitimate research purposes will be subject to the Merck KGaA, Darmstadt, Germany Data Sharing Policy. All requests should be submitted in writing to the Merck KGaA, Darmstadt, Germany data-sharing portal (https://www.merckgroup.com/en/research/our-approach-to-research-and-development/healthcare/clinicaltrials/commitment-responsible-data-sharing.html). When Merck KGaA, Darmstadt, Germany has a co-research, co-development, or co-marketing or co-promotion agreement, or when the product has been out-licensed, the responsibility for disclosure might be dependent on the agreement between parties. Under these circumstances, Merck KGaA, Darmstadt, Germany, will endeavour to gain agreement to share data in response to requests.
