# Supplementary material for: A first-in-man phase 1 study of the DNA-dependent protein kinase inhibitor peposertib (formerly M3814) in patients with advanced solid tumours
Source: Br J Cancer. 2020 Nov 24;124(4):728–35. doi: 10.1038/s41416-020-01151-6 (PMC7884679; doi:10.1038/s41416-020-01151-6)
Supplement: Supplementary file 1 — Supplementary material [file 41416_2020_1151_MOESM1_ESM.docx]

## Supplementary material

### **Supplementary Fig. S1.** Study schematic

Description: After screening (21 days), patients received peposertib (formerly M3814) in 3-week (21‑day) cycles. Following treatment discontinuation, patients completed an end of treatment visit. A follow‑up period continued for 30 (± 3) days after the last dose of peposertib. Patients completed an end of trial visit at the end of the follow‑up period.


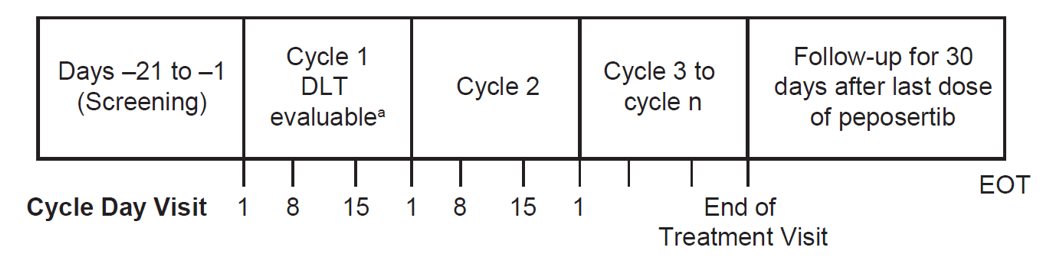


^a^The DLT observation period was 21 days of the first cycle of treatment. DLTs were evaluated in order to make decisions regarding dose escalation or evaluation of intermediate dose levels.

EOT, end of trial visit.

### **Supplementary Table S1.** Overall summary of TEAEs

Description: The total number of TEAEs by dose cohort (Safety Analysis Set) is presented in Supplementary Table S1.

| **Number of patients, *n* (%), with:** | **Peposertib^a^ dose (mg)/frequency of administration** | | | | | | | **Total**  ***N*= 31** |
| --- | --- | --- | --- | --- | --- | --- | --- | --- |
|  | **100/QD**  ***n* = 3** | **200/QD**  ***n* = 3** | **150/BID**  ***n* = 3** | **200/BID**  ***n* = 3** | **300/BID**  ***n* = 9** | **400/BID**  ***n* = 4** | **400/BID (RP2D)**  ***n* = 6** |  |
| Any TEAE | 3 (100) | 3 (100) | 3 (100) | 3 (100) | 9 (100) | 4 (100) | 6 (100) | 31 (100) |
| Any peposertib-related TEAE | 1 (33) | 2 (67) | 2 (67) | 0 (0) | 8 (89) | 3 (75) | 6 (100) | 22 (71) |
| Any serious TEAE | 1 (33) | 1 (33) | 2 (67) | 1 (33) | 5 (56) | 3 (75) | 4 (67) | 17 (55) |
| Any peposertib-related serious TEAE | 0 (0) | 0 (0) | 0 (0) | 0 (0) | 0 (0) | 1 (25) | 3 (50) | 4 (13) |
| Any Grade ≥3 TEAE | 2 (67) | 1 (33) | 3 (100) | 2 (67) | 5 (56) | 3 (75) | 5 (83) | 21 (68) |
| Any Grade ≥4 TEAE | 0 (0) | 0 (0) | 0 (0) | 0 (0) | 0 (0) | 2 (50)^b^ | 0 (0) | 2 (6) |
| Any peposertib-related Grade ≥3 TEAE | 0 (0) | 0 (0) | 0 (0) | 0 (0) | 3 (33) | 1 (25) | 3 (50) | 7 (23) |
| Any peposertib-related Grade ≥4 TEAE | 0 (0) | 0 (0) | 0 (0) | 0 (0) | 0 (0) | 0 (0) | 0 (0) | 0 (0) |
| Any TEAE leading to study discontinuation | 0 (0) | 0 (0) | 0 (0) | 0 (0) | 1 (11) | 1 (25) | 2 (33) | 4 (13) |
| Any TEAE leading to death | 0 (0) | 0 (0) | 0 (0) | 0 (0) | 0 (0) | 1 (25) | 0 (0) | 1 (3) |
| Any peposertib related TEAE leading to death | 0 (0) | 0 (0) | 0 (0) | 0 (0) | 0 (0) | 0 (0) | 0 (0) | 0 (0) |

^a^Formerly M3814; ^b^AEs of blood alkaline phosphatase increased were not considered a TEAE for one patient (occurred prior to the start of study treatment).

BID, twice daily; QD, once daily; RP2D, recommended phase II dose; TEAE, treatment-emergent adverse event.

### **Supplementary Table S2.** Key summary statistics of peposertib^a^ PK parameters by dose group on Cycle 1 Day 1 and Cycle 2 Day 1 (PK Analysis Set)

Description: Pharmacokinetic parameters following single and multiple doses of peposertib are summarised in Supplementary Table S2.

| **Peposertib dose group** |  | **C_max_**  **(ng/mL)** | **t_max_**  **(h)** | **AUC_0–∞_**  **(h*ng/mL)^b^** | **AUC_0–12_**  **(h*ng/mL)** | **CL_/f_**  **(L/h)^b^** | **V_z/f_**  **(L)^b^** | **t_1/2_**  **(h)^b^** | **C_max_/dose**  **(ng/mL/mg)** | **AUC_0–12_/dose**  **([h*ng/mL]/mg)** | **R_acc_ C_max_** | **R_acc_ AUC_0–12_** |
| --- | --- | --- | --- | --- | --- | --- | --- | --- | --- | --- | --- | --- |
| **Cycle 1 Day 1** | | | | | | | | | | | | |
| **100 mg QD** | ***n*** | 3 | 3 | 3 | 3 | 3 | 3 | 3 | 3 | 3 | – | – |
|  | **Mean** | 561 | 1.7 | 2,920 | 2,220 | 54.2 | 508 | 6.6 | 5.6 | 22.2 | – | – |
|  | **Min** | 161 | 1.5 | 908 | 670 | 21.8 | 194 | 4.4 | 1.6 | 6.7 | – | – |
|  | **Median** | 512 | 1.6 | 3,270 | 2,730 | 30.6 | 273 | 6.7 | 5.1 | 27.3 | – | – |
|  | **Max** | 1,010 | 2.1 | 4,590 | 3,250 | 110 | 1,060 | 8.7 | 10.1 | 32.5 | – | – |
|  | **GeoMean** | 437 | 1.7 | 2,390 | 1,810 | 41.9 | 382 | 6.3 | 4.4 | 18.1 | – | – |
| **200 mg QD** | ***n*** | 3 | 3 | 3 | 3 | 3 | 3 | 3 | 3 | 3 | – | – |
|  | **Mean** | 1,490 | 1.4 | 9,970 | 6,080 | 40.2 | 433 | 9.4 | 7.4 | 30.4 | – | – |
|  | **Min** | 539 | 1.1 | 2,880 | 2,190 | 9.0 | 191 | 6.1 | 2.7 | 10.9 | – | – |
|  | **Median** | 1,070 | 1.5 | 4,730 | 3,870 | 42.3 | 369 | 7.4 | 5.4 | 19.3 | – | – |
|  | **Max** | 2,850 | 1.5 | 22,300 | 12,200 | 69.3 | 741 | 14.7 | 14.3 | 60.9 | – | – |
|  | **GeoMean** | 1,180 | 1.3 | 6,730 | 4,690 | 29.7 | 374 | 8.7 | 5.9 | 23.4 | – | – |
| **150 mg BID** | ***n*** | 3 | 3 | 3 | 3 | 3 | 3 | 3 | 3 | 3 | – | – |
|  | **Mean** | 695 | 1.3 | 3,770 | 3,040 | 40.5 | 304 | 5.1 | 4.6 | 20.3 | – | – |
|  | **Min** | 330 | 1.0 | 3,320 | 2,170 | 33.0 | 202 | 3.5 | 2.2 | 14.5 | – | – |
|  | **Median** | 594 | 1.5 | 3,460 | 3,180 | 43.3 | 218 | 4.2 | 4.0 | 21.2 | – | – |
|  | **Max** | 1,160 | 1.5 | 4,550 | 3,770 | 45.2 | 492 | 7.6 | 7.7 | 25.2 | – | – |
|  | **GeoMean** | 610 | 1.3 | 3,740 | 2,960 | 40.1 | 279 | 4.8 | 4.1 | 19.8 | – | – |
| **200 mg BID** | ***n*** | 3 | 3 | 3 | 3 | 3 | 3 | 3 | 3 | 3 | – | – |
|  | **Mean** | 393 | 2.0 | 1,780 | 1,370 | 143 | 1,010 | 5.6 | 2.0 | 6.9 | – | – |
|  | **Min** | 192 | 1.0 | 787 | 700 | 81.8 | 442 | 3.3 | 1.0 | 3.5 | – | – |
|  | **Median** | 260 | 1.1 | 2,120 | 1,420 | 94.3 | 1,120 | 4.0 | 1.3 | 7.1 | – | – |
|  | **Max** | 727 | 4.0 | 2,440 | 2,000 | 254 | 1,480 | 9.5 | 3.6 | 10.0 | – | – |
|  | **GeoMean** | 331 | 1.7 | 1,600 | 1,260 | 125 | 901 | 5.0 | 1.7 | 6.3 | – | – |
| **300 mg BID** | ***n*** | 9 | 9 | 9 | 9 | 9 | 9 | 9 | 9 | 9 | – | – |
|  | **Mean** | 839 | 1.9 | 6,050 | 4,270 | 127 | 662 | 4.7 | 2.8 | 14.2 | – | – |
|  | **Min** | 156 | 1.0 | 781 | 702 | 14.4 | 182 | 2.0 | 0.5 | 2.3 | – | – |
|  | **Median** | 677 | 1.5 | 2,290 | 2,250 | 131 | 425 | 4.6 | 2.3 | 7.5 | – | – |
|  | **Max** | 2,070 | 4.0 | 20,800 | 12,400 | 384 | 1,750 | 8.8 | 6.9 | 41.5 | – | – |
|  | **GeoMean** | 654 | 1.7 | 3,690 | 2,950 | 81.4 | 489 | 4.2 | 2.2 | 9.9 | – | – |
| **400 mg BID** | ***n*** | 4 | 4 | 4 | 4 | 4 | 4 | 4 | 4 | 4 | – | – |
|  | **Mean** | 717 | 2.4 | 4,140 | 3,580 | 172 | 511 | 3.0 | 1.8 | 8.9 | – | – |
|  | **Min** | 451 | 0.5 | 1,020 | 1,020 | 44.5 | 290 | 1.3 | 1.1 | 2.6 | – | – |
|  | **Median** | 493 | 2.5 | 3,280 | 2,920 | 126 | 517 | 3.1 | 1.2 | 7.3 | – | – |
|  | **Max** | 1,430 | 4.0 | 8,990 | 7,450 | 391 | 720 | 4.5 | 3.6 | 18.6 | – | – |
|  | **GeoMean** | 629 | 1.9 | 3,130 | 2,830 | 128 | 479 | 2.6 | 1.6 | 7.1 | – | – |
| **Cycle 2 Day 1 (clearance is CL_ss_)** | | | | | | | | | | | | |
| **100 mg QD** | ***n*** | 3 | 3 | – | 3 | 3 | 3 | 3 | 3 | 3 | 3 | 3 |
|  | **Mean** | 440 | 1.8 | – | 2,290 | 39.3 | 276 | 5.5 | 4.4 | 22.9 | 1.3 | 1.4 |
|  | **Min** | 390 | 1.5 | – | 1,630 | 24.3 | 241 | 2.9 | 3.9 | 16.3 | 0.4 | 0.7 |
|  | **Median** | 408 | 1.6 | – | 2,130 | 36.2 | 264 | 6.2 | 4.1 | 21.3 | 1.0 | 1.1 |
|  | **Max** | 523 | 2.4 | – | 3,100 | 57.3 | 322 | 7.5 | 5.2 | 31.0 | 2.4 | 2.4 |
|  | **GeoMean** | 437 | 1.8 | – | 2,210 | 36.9 | 274 | 5.1 | 4.4 | 22.1 | 1.0 | 1.2 |
| **200 mg QD** | ***n*** | 3 | 3 | – | 3 | 3 | 3 | 3 | 3 | 3 | 3 | 3 |
|  | **Mean** | 1,190 | 1.8 | – | 6,520 | 30.8 | 198 | 4.5 | 5.9 | 32.6 | 0.9 | 1.4 |
|  | **Min** | 654 | 1.1 | – | 4,360 | 15.7 | 109 | 4.1 | 3.3 | 21.8 | 0.7 | 0.9 |
|  | **Median** | 931 | 1.5 | – | 4,630 | 37.8 | 231 | 4.7 | 4.7 | 23.1 | 0.9 | 1.2 |
|  | **Max** | 1,970 | 3.0 | – | 10,600 | 38.9 | 255 | 4.8 | 9.9 | 52.9 | 1.2 | 2.0 |
|  | **GeoMean** | 1,060 | 1.7 | – | 5,970 | 28.5 | 186 | 4.5 | 5.3 | 29.9 | 0.9 | 1.3 |
| **150 mg BID** | ***n*** | 3 | 3 | – | 3 | 3 | 3 | 3 | 3 | 3 | 3 | 3 |
|  | **Mean** | 699 | 2.7 | – | 5,000 | 30.3 | 294 | 6.8 | 4.7 | 33.3 | 1.3 | 1.8 |
|  | **Min** | 586 | 2.0 | – | 4,350 | 27.0 | 242 | 5.7 | 3.9 | 29.0 | 0.6 | 1.2 |
|  | **Median** | 720 | 2.1 | – | 5,090 | 29.5 | 314 | 6.3 | 4.8 | 33.9 | 1.0 | 1.6 |
|  | **Max** | 792 | 4.0 | – | 5,560 | 34.5 | 327 | 8.4 | 5.3 | 37.1 | 2.4 | 2.6 |
|  | **GeoMean** | 694 | 2.6 | – | 4,970 | 30.2 | 292 | 6.7 | 4.6 | 33.1 | 1.1 | 1.7 |
| **200 mg BID** | ***n*** | 3 | 3 | – | 3 | 3 | 3 | 3 | 3 | 3 | 3 | 3 |
|  | **Mean** | 671 | 2.1 | – | 3,320 | 74.4 | 447 | 4.4 | 3.4 | 16.6 | 2.8 | 2.9 |
|  | **Min** | 343 | 1.4 | – | 1,730 | 37.1 | 267 | 3.9 | 1.7 | 8.7 | 0.5 | 0.9 |
|  | **Median** | 599 | 2.1 | – | 2,830 | 70.7 | 431 | 4.2 | 3.0 | 14.1 | 2.3 | 3.8 |
|  | **Max** | 1,070 | 3.0 | – | 5,400 | 115 | 644 | 5.0 | 5.4 | 27.0 | 5.6 | 4.0 |
|  | **GeoMean** | 604 | 2.0 | – | 2,980 | 67.1 | 420 | 4.3 | 3.0 | 14.9 | 1.8 | 2.4 |
| **300 mg BID** | ***n*** | 5 | 5 | – | 5 | 5 | 5 | 5 | 5 | 5 | 5 | 5 |
|  | **Mean** | 1,730 | 1.7 | – | 11,900 | 51.1 | 448 | 6.21 | 5.8 | 39.8 | 2.7 | 4.2 |
|  | **Min** | 507 | 1.0 | – | 3,580 | 8.2 | 88.0 | 3.71 | 1.7 | 11.9 | 1.5 | 1.9 |
|  | **Median** | 1,000 | 2.0 | – | 6,180 | 48.6 | 394 | 5.62 | 3.3 | 20.6 | 2.4 | 3.4 |
|  | **Max** | 4,870 | 2.1 | – | 36,800 | 83.8 | 1,060 | 8.79 | 16.2 | 123 | 4.9 | 7.6 |
|  | **GeoMean** | 1,260 | 1.7 | – | 7,750 | 38.7 | 333 | 5.95 | 4.2 | 25.8 | 2.4 | 3.8 |
| **400 mg BID** | ***n*** | 6 | 6 | – | 6 | 6 | 6 | 6 | 6 | 6 | 3 | 3 |
|  | **Mean** | 1,930 | 1.5 | – | 12,800 | 50.1 | 252 | 4.7 | 4.8 | 31.9 | 1.9 | 3.2 |
|  | **Min** | 730 | 0.0^c^ | – | 2,770 | 18.4 | 128 | 2.0 | 1.8 | 6.9 | 1.5 | 2.5 |
|  | **Median** | 2,060 | 1.7 | – | 13,100 | 31.0 | 233 | 5.4 | 5.1 | 32.8 | 1.6 | 2.7 |
|  | **Max** | 3,050 | 2.1 | – | 21,800 | 144 | 425 | 6.1 | 7.6 | 54.4 | 2.7 | 4.4 |
|  | **GeoMean** | 1,740 | NC | – | 10,600 | 37.9 | 235 | 4.3 | 4.4 | 26.4 | 1.9 | 3.1 |

^a^Formerly M3814; ^b^Diagnostic parameters indicated that λz and thus derived PK parameters could not be precisely estimated for most patients; ^c^Pre-dose sample assigned a time of 0 h.

AUC_0–12_, area under the plasma concentration–time curve from 0 to 12 h; AUC_0–∞_, area under the plasma concentration–time curve from 0 to infinity; BID, twice daily; C_L/f_, total body clearance of drug; C_Lss_, clearance at steady state of drug; C_max_, maximum observed concentration; GeoMean, geometric mean; max, maximum; min, minimum; NC, not calculated; PK, pharmacokinetic; QD, once daily; R_acc_ AUC_0–12_, accumulation ratio for AUC_0–12_; R_acc_ C_max_, accumulation ratio for C_max_; t_1/2_, elimination half-life; V_z/f_, apparent volume of distribution during terminal phase.
